# Serum Uric Acid as a Mediator of Insulin Resistance: Molecular Mechanisms and Metabolic Pathways

**DOI:** 10.1002/edm2.70163

**Published:** 2026-01-11

**Authors:** Nurshad Ali

**Affiliations:** ^1^ Department of Biochemistry and Molecular Biology Shahjalal University of Science and Technology Sylhet Bangladesh

**Keywords:** insulin resistance, mechanisms, pathways, serum uric acid

## Abstract

**Background:**

Insulin resistance (IR) is a key factor in metabolic conditions such as type 2 diabetes (T2D) and metabolic syndrome, which significantly impact global health. Serum uric acid (SUA), is the end product of purine catabolism, has increasingly been recognized as a potential modulator of insulin sensitivity.

**Methods:**

A comprehensive narrative review was conducted to synthesize current evidence on SUA–mediated insulin resistance, with a focus on underlying molecular mechanisms, clinical implications, and key gaps warranting future investigation. Relevant experimental, translational, and clinical studies examining the role of SUA in insulin resistance, its mechanistic pathways, and therapeutic potential were critically analysed.

**Results:**

Emerging evidence indicates that elevated SUA levels are associated with disturbances in insulin signaling pathways. Mechanistically, high SUA levels can lead to oxidative stress, endothelial dysfunction, inflammation, and impaired function of adipocytes—all of which collectively impede insulin receptor activity and downstream signaling. Key pathways involved include activation of the NLRP3 inflammasome, suppression of AMP‐activated protein kinase (AMPK), and induction of mitochondrial dysfunction. These mechanisms contribute to altered insulin sensitivity in both hepatic and adipose tissues. Clinically, higher SUA levels are associated with increased risk of developing metabolic syndrome, T2D, and cardiovascular diseases, highlighting SUA's potential as both a biomarker and a therapeutic target. Despite these findings, the precise molecular interactions between SUA and insulin signaling remain incompletely understood, underscoring the need for further translational and mechanistic research.

## Graphical abstract

Elevated SUA can lead to oxidative stress, endothelial dysfunction, inflammation and impaired function of adipocytes—all of which collectively impede insulin receptor activity and downstream signalling. Key pathways involved include activation of the NLRP3 inflammasome, suppression of AMPK and induction of mitochondrial dysfunction. These mechanisms contribute to altered insulin sensitivity in both hepatic and adipose tissues.

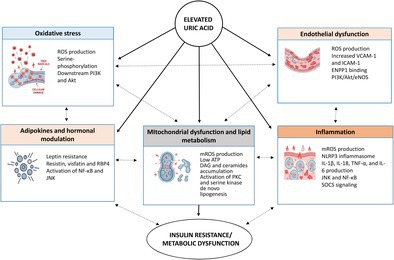

## 1 Introduction

Insulin resistance (IR) is a condition in which cells have a reduced response to insulin, resulting in decreased glucose uptake and increased blood glucose levels. It is a key factor in the onset of metabolic diseases such as type 2 diabetes (T2D), metabolic syndrome and cardiovascular diseases [1]. The incidence of IR worldwide is increasing, with studies revealing that about 40% of adults in the United States are affected, a pattern that aligns with the rising rates of obesity and sedentary lifestyles [2].

SUA, which has been traditionally linked to gout and kidney stones, has also gained interest for its possible effects on metabolic health. High levels of SUA, known as hyperuricemia, have been associated with the development of various metabolic conditions [3]. Recent findings indicate that SUA might affect insulin sensitivity through several mechanisms, including the induction of oxidative stress, inflammation and damage to endothelial function [4].

Epidemiological studies have identified a significant relationship between hyperuricemia and IR. For example, a study analysed data from the NHANES found that increased SUA levels are positively associated with the likelihood of metabolic syndrome in both men and women [5]. Moreover, findings suggest that SUA levels can serve as predictors for the development of hypertension, diabetes, obesity and kidney issues, highlighting their significance in metabolic health [6].

Understanding the molecular processes that connect SUA to IR is vital for crafting targeted treatment approaches. Although the specific pathways are still being explored, existing research suggests that SUA might disrupt insulin signalling through various routes, such as by activating the NLRP3 inflammasome and inhibiting AMP‐activated protein kinase (AMPK) [4]. This review intends to clarify the molecular mechanisms through which SUA influences IR, with an emphasis on important metabolic pathways and their consequences for disease advancement. By integrating current research findings, we aim to offer a thorough understanding of SUA's involvement in metabolic disorders and point out promising areas for future investigations and therapeutic development.

## 2 Uric Acid Metabolism and Homeostasis

**FIGURE 1:**
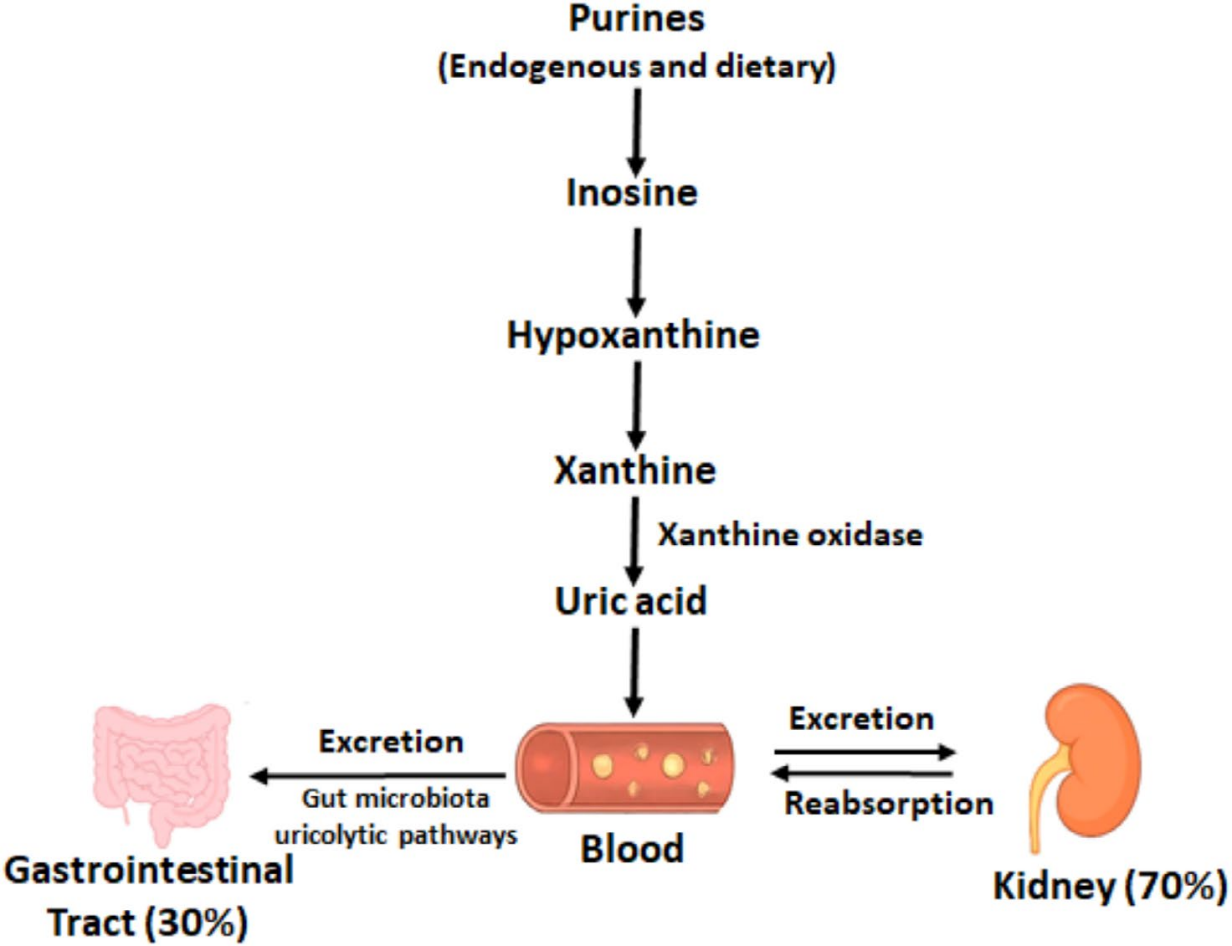
Overview of uric acid metabolism and homeostasis.

An overview of purine metabolism and homeostasis is presented in Figure 1. Uric acid (UA) is the final oxidation product of purine degradation in humans. Purines, which come from both dietary intake and endogenous nucleotide turnover, are progressively broken down into hypoxanthine and xanthine, which are converted to UA by xanthine oxidoreductase (XOR) [7]. XOR exists in two interchangeable forms: xanthine dehydrogenase (XDH) and xanthine oxidase (XO) [7, 8]. Under normal conditions, XDH predominates and uses NAD^+^ as an electron acceptor, which produces NADH. However, during oxidative stress, tissue injury, or inflammation, XDH is changed to XO. XO uses molecular oxygen as an electron acceptor, creating reactive oxygen species (ROS) like superoxide and hydrogen peroxide. This shift increases oxidative stress and leads to metabolic and vascular dysfunction.

Humans lack uricase, the enzyme that converts UA to allantoin, leading to higher circulating UA levels than in most mammals. The majority of UA production takes place in the liver, intestines, and vascular endothelium. Homeostasis depends on a balance between production and excretion. About two‐thirds of UA is eliminated via the kidneys, while the remaining one‐third is excreted through the gastrointestinal tract via gut microbiota's uricolytic pathways [7, 9]. Renal elimination consists of glomerular filtration, tubular reabsorption (mainly via URAT1 and GLUT9), secretion, and post‐secretory absorption, facilitated by transporters including ABCG2. Genetics variants (e.g., SLC2A9, ABCG2) in these transporters significantly alter the excretory pathways and can lead to hyperuricemia [6].

Dietary purines, fructose, and alcohol increase SUA levels. Fructose speeds up ATP breakdown, while alcohol boosts lactate‐related URAT1 reabsorption [10]. Metabolic conditions such as obesity and IR enhance renal UA reabsorption through insulin‐mediated effects on URAT1 and GLUT9 [10]. Reduced kidney function and certain medications (e.g., diuretics) further impair UA excretion.

Typical SUA levels are between 3.5–7.2 mg/dL in men and 2.6–6.0 mg/dL in women. Levels exceeding these limits are diagnosed as hyperuricemia, which is linked to gout and other metabolic disorders including hypertension and cardiovascular issues [6]. Furthermore, levels outside the normal range influence redox balance, as UA functions as both an antioxidant and a pro‐oxidant depending on the biochemical context [11].

## 3 Molecular Mechanisms Linking SUA and Insulin Resistance

**FIGURE 2:**
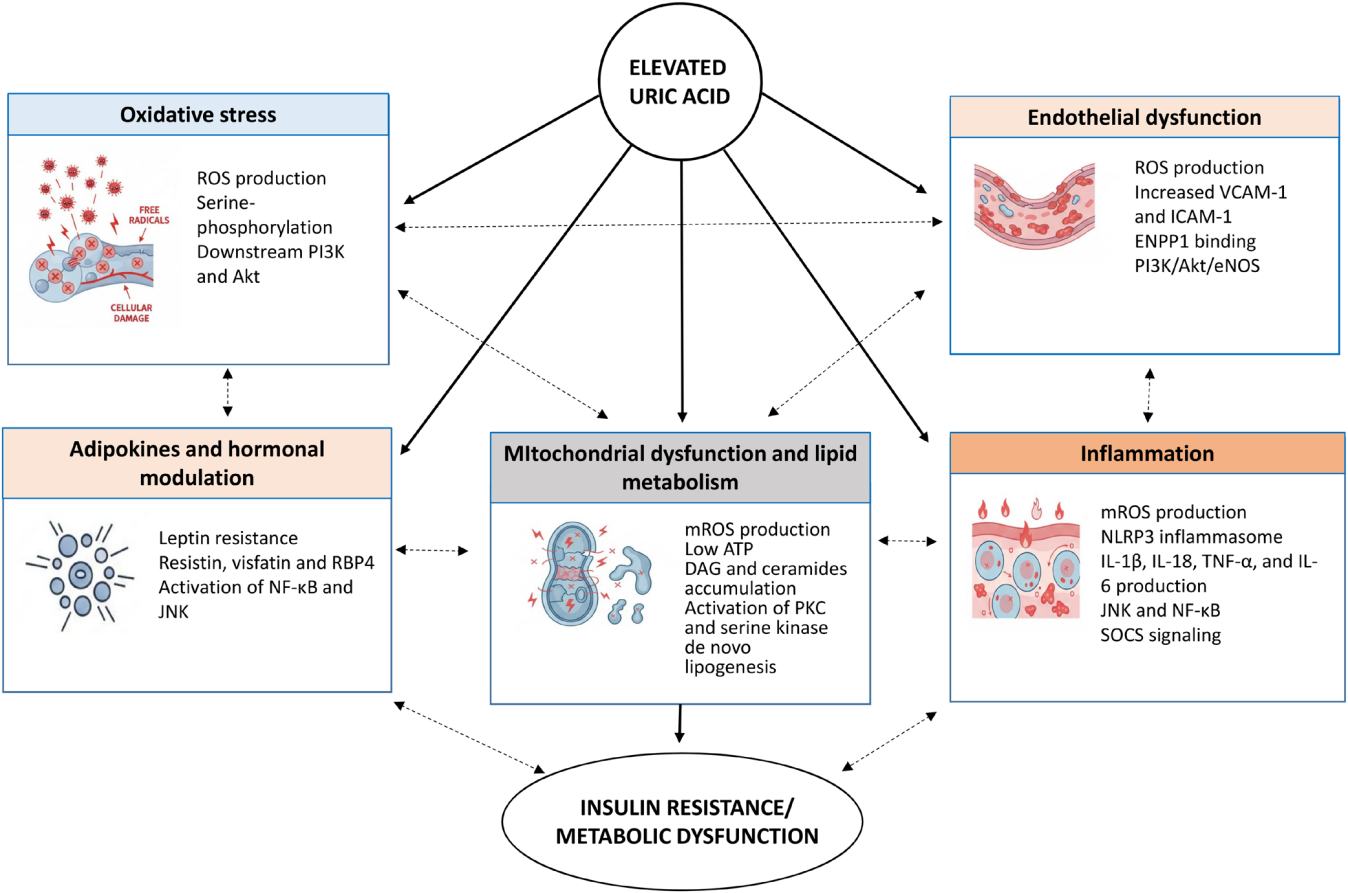
Molecular pathways linking serum uric acid to insulin resistance.

The emerging hypothesis that elevated SUA contributes to the development of IR is supported by mechanistic evidence involving oxidative stress, endothelial dysfunction, inflammation, mitochondrial abnormalities, and altered adipose tissue signalling, as discussed in the following sections and summarised in Table 1 and Figure 2.

**TABLE 1:** Key molecular mechanisms linking elevated serum uric acid to insulin resistance.

Mechanistic pathway	Key molecular events	Impact on insulin signalling/metabolism	References
Oxidative stress	ROS generation during xanthine oxidase activity; activation of stress kinases (JNK, p38 MAPK)	Serine phosphorylation of IRS‐1; impaired PI3K/Akt signalling	[12, 13]
Endothelial dysfunction	Reduced NO bioavailability; eNOS uncoupling; ENPP1 recruitment to insulin receptor	Vascular insulin resistance; reduced insulin‐mediated glucose delivery to muscle	[14, 15]
Inflammation	NLRP3 inflammasome activation; IL‐1β and IL‐18 release; NF‐κB activation	Cytokine‐mediated IRS‐1 inhibition; SOCS induction	[16, 17]
Mitochondrial dysfunction and lipid metabolism	SUA‐induced mitochondrial ROS; reduced ATP synthesis; impaired fatty acid oxidation	Lipotoxicity, ectopic fat deposition; PKC activation interfering with insulin signalling	[18]
Adipokines & Hormonal Modulation	Decreased adiponectin; altered leptin sensitivity; dysregulation of other adipokines (resistin, RBP4)	Reduced adipose insulin sensitivity; increased systemic IR	[18, 19]

*Note:* The principal molecular mechanisms by which elevated SUA contributes to IR are summarised in the table.

Abbreviations: eNOS, endothelial nitric oxide synthase; ENPP1, ectonucleotide pyrophosphatase/phosphodiesterase 1; IRS‐1, insulin receptor substrate‐1; NO, nitric oxide; PKC, protein kinase C; RBP4, retinol‐binding protein 4; ROS, reactive oxygen species; SOCS, suppressor of cytokine signalling.

### 3.1 Oxidative Stress

ROS are produced during the oxidation of xanthine by the enzyme XOR, which generates UA. When XO is favoured over XDH, a higher production of superoxide and hydrogen peroxide occurs alongside the synthesis of UA. Some laboratory and animal studies indicate that inhibiting XOR can reduce oxidative stress and increase insulin sensitivity, as seen with medications like allopurinol or febuxostat [12, 20]. Furthermore, clinical evidence suggests that SUA levels correlate with markers of oxidative stress, regardless of XOR activity, implying that UA might also contribute to ROS production or diminish antioxidant levels [13]. Excessive ROS can disrupt insulin signalling by encouraging serine phosphorylation instead of tyrosine phosphorylation of insulin receptor substrates (IRS), hindering their interaction with the insulin receptor and downstream mediators like phosphoinositide 3‐kinase (PI3K) and protein kinase B (Akt) [4, 21]. An increase in ROS can also trigger stress kinases, such as JNK and p38 MAPK, as well as phosphatases, including protein tyrosine phosphatases (PTPs), which dephosphorylate molecules such as IRS‐1 or Akt, thereby diminishing glucose uptake stimulated by insulin. In hyperuricemia, these oxidative changes may worsen IR in muscle, liver, or adipose tissues [4].

### 3.2 Endothelial Dysfunction

Endothelial cells play an important role in regulating vascular tone and insulin‐mediated glucose delivery. A key mediator of this process is nitric oxide (NO), which is produced by endothelial nitric oxide synthase (eNOS) from L‐arginine. NO promotes vasodilation, improves microvascular blood flow, and facilitates insulin and glucose delivery to skeletal muscle and other tissues [22].

High levels of SUA reduce NO availability through various ways. SUA increases oxidative stress, which leads to the production of ROS like superoxide. Superoxide quickly reacts with NO to form peroxynitrite, which decreases the amount of functional NO. Moreover, SUA can lead to eNOS uncoupling, where eNOS produces superoxide instead of NO. This further decreases NO production and impairs vasodilation [14]. Hyperuricemia also disrupts microvascular integrity by increasing endothelial activation. This is marked by higher levels of adhesion molecules like Vascular Cell Adhesion Molecule‐1 (VCAM‐1) and Intercellular Cell Adhesion Molecule‐1 (ICAM‐1), along with a decrease in the microvascular density [23].

At the molecular level, UA affects insulin receptors function in endothelial cells. In human umbilical vein endothelial cells (HUVECs), UA promotes the binding of ectonucleotide pyrophosphatase/phosphodiesterase 1 (ENPP1) to the insulin receptor [15]. This binding blocks signalling through the PI3K/Akt/eNOS pathway. As a result, NO production drops, and insulin‐mediated vasodilation decreases, a phenomenon known as vascular insulin resistance. Importantly, blocking URAT1, a UA transporter, can reverse these effects, highlighting the direct impact of intracellular UA on endothelial dysfunction [4, 15].

### 3.3 Inflammation

A major inflammatory process connecting high SUA levels to insulin resistance is the activation of the NLR Family Pyrin Domain Containing 3 (NLRP3) inflammasome. The NLRP3 inflammasome is a protein complex that detects stress signals in cells and triggers the production of inflammatory cytokines. When SUA levels become too high or form monosodium urate (MSU) crystals, it acts as a danger‐associated molecular pattern (DAMP), starting a series of events [16].

Step 1 (Priming, Signal 1): Pattern‐recognition receptors, like Toll‐like receptors, activate nuclear factor‐κB (NF‐κB). This increases the production of NLRP3 and the inactive forms of pro‐IL‐1β and pro‐IL‐18 [17]. Step 2 (Activation, Signal 2): The uptake of UA or MSU crystals inside cells leads to the creation of mitochondrial ROS, ionic flux (including K^+^ efflux), and disruption of lysosomes. These events promote the assembly of the NLRP3 inflammasome [17]. Step 3 (Caspase‐1 activation): The assembled inflammasome brings in and activates caspase‐1. This enzyme breaks down pro‐IL‐1β and pro‐IL‐18 into their active forms, which can then be released [17]. Step 4 (Cytokine‐mediated impairment of insulin signalling): Released IL‐1β, IL‐18, TNF‐α, and IL‐6 activate inflammatory pathways like c‐Jun N‐terminal kinase (JNK) and NF‐κB. This leads to the phosphorylation of insulin receptor substrate (IRS) proteins, interfering with normal insulin signalling. Chronic inflammation also promotes suppressor of cytokine signalling (SOCS) proteins that break down IRS or prevent its phosphorylation, further disrupting insulin function.

Animal studies show that mice without NLRP3 or treated with NLRP3 inhibitors show better insulin sensitivity during metabolic stress, highlighting the importance of this pathway [24].

### 3.4 Mitochondrial Dysfunction and Lipid Metabolism

Elevated SUA may impair insulin sensitivity through several connected mitochondrial and lipid‐metabolism pathways. Normally, mitochondria maintain energy balance in cells by breaking down substrates and producing ATP using the electron transport chain (ETC). When SUA levels increase, UA can enter cells and directly disrupt mitochondrial function. This happens by enhancing mitochondrial superoxide production and reducing ETC activity, especially at complexes I and III [25]. This disruption lowers ATP production and raises oxidative stress, creating an environment inside cells that makes them less responsive to insulin, particularly in energy‐demanding tissues like skeletal muscle. Decreased ATP availability and increased ROS also lead to metabolic inflexibility. In this condition, cells have trouble switching efficiently between using carbohydrates and fatty acids for energy [26].

Mitochondrial dysfunction further disrupts lipid handling by lowering the capacity for fatty‐acid β‐oxidation, leading to the accumulation of lipotoxic intermediates like diacylglycerols and ceramides. These substances activate certain protein kinase C (PKC) isoforms and serine kinases that add phosphate groups to insulin receptor substrates at sites, thereby inhibiting insulin signalling [18]. In liver cells, SUA‐induced mitochondrial oxidative stress causes steatosis and enhances de novo lipogenesis, partly by upregulating lipogenic enzymes [25]. Furthermore, UA may impair adipocyte lipid‐storage capacity, leading to a ‘spillover’ of fatty acids into insulin‐sensitive tissues. Together, the effects of mitochondrial ROS, lipid accumulation, and kinase activation form a coherent mechanistic pathway that links high SUA levels with insulin resistance in the liver and skeletal muscles.

### 3.5 Adipokines and Hormonal Modulation

Adipose tissue acts as an active endocrine organ, releasing adipokines that tightly regulate whole‐body insulin sensitivity. High levels of SUA can disrupt the expression and signalling of these hormones, leading to IR. One well‐known effect is the way UA suppresses adiponectin, an insulin‐sensitising adipokine. Adiponectin improves fatty acid oxidation and glucose uptake through AMPK activation. When adiponectin levels drop, these metabolic pathways weaken, and inflammation in adipose tissue increases, promoting systemic IR [27].

UA also affects leptin signalling, which hinders leptin's role in managing appetite, lipid mobilisation, and insulin sensitivity. Elevated UA levels may lead to leptin resistance by disrupting leptin receptor signalling and increasing the production of inflammatory cytokines in adipose tissue, worsening metabolic dysfunction [27]. In addition to adiponectin and leptin, SUA may influence other adipokines, such as resistin, visfatin, and retinol‐binding protein 4 (RBP4). Each of these molecules plays a role in insulin signalling. Resistin activates inflammatory pathways like NF‐κB and JNK. Visfatin is involved in NAD^+^ biosynthesis and metabolic control [28]. RBP4 reduces insulin‐stimulated glucose uptake in muscle and boosts glucose production in the liver [28].

Since adipose tissue controls systemic lipid flow, changes in the balance of adipokines and insulin signalling due to SUA can impact areas beyond adipose tissue, contributing to whole‐body IR [4, 28].

## 4 Crosstalk With Other Metabolic Pathways

SUA interacts with various metabolic pathways, and many of these effects are built on the upstream mechanisms described in earlier sections—inclusive of UA–induced oxidative stress, mitochondrial dysfunction, and inflammation. These foundational processes provide the basis for how SUA influences glucose and lipid homeostasis.

Elevated SUA has been linked to enhanced hepatic gluconeogenesis and reduced glucose uptake in peripheral tissues by activating oxidative stress–responsive kinases such as JNK and p38 MAPK. These pathways lead to serine phosphorylation of insulin receptor substrate‐1 (IRS‐1), which in turn reduces PI3K/Akt signalling and hinders glucose transport in skeletal muscle and adipose tissues [12]. In liver cells, SUA has been found to increase the expression of sterol regulatory element‐binding protein‐1c (SREBP‐1c), promoting de novo lipogenesis and the accumulation of triglycerides [29]. Together, these alterations promote ectopic fat deposition, liver steatosis, and downstream insulin resistance, which lead to metabolic imbalance.

Epidemiological studies consistently show a strong relationship between SUA levels and the various components of metabolic syndrome, including central obesity, elevated blood pressure, high triglyceride levels, and abnormal glucose metabolism [3, 5, 18]. Data from both cross‐sectional and longitudinal studies suggest that increased SUA levels often precede the onset of IR related to obesity and T2D [30, 31].

Experimental studies indicate that hyperuricemia caused by fructose consumption plays a role in weight gain by promoting liver fat accumulation and reducing energy expenditure [29]. On the other hand, obesity tends to hinder the kidneys' ability to excrete UA due to increased sodium reabsorption and the upregulation of urate transport mechanisms, which further raises SUA levels [19].

Thus, SUA serves as both a driver and a downstream consequence of metabolic dysregulation. Elevated SUA levels can lead to IR, liver fat accumulation, and inflammation in adipose tissue, while metabolic issues—mainly obesity and high insulin levels—can decrease the renal clearance of UA, reinforcing hyperuricemia [32]. This reciprocal relationship creates a self‐sustaining cycle where SUA acts as both a mediator and an indicator of metabolic syndrome. Understanding this dual interaction highlights the importance of comprehensive treatment strategies that address both SUA levels and broader metabolic risk factors.

## 5 Evidence From Experimental and Clinical Studies

To support the mechanistic framework described in the previous sections, this part summarises the key findings from both pre‐clinical and human studies that connect SUA to IR and broader metabolic dysfunction.

### 5.1 Experimental and Animal Studies

Animal studies have shown strong evidence that elevated SUA can lead to the development of IR (Table 2). Rodents that are given high‐fructose diets develop elevated UA levels along with disturbances in insulin signalling, liver fat accumulation, and weight gain. The use of drugs that inhibit xanthine oxidase, such as allopurinol or febuxostat, in these models results in reduced SUA and helps to prevent the development of IR and characteristics of metabolic syndrome [4, 29].

**TABLE 2:** Key experimental and clinical evidence linking serum uric acid to insulin resistance.

Model/Population	Design and intervention	Major findings	References
C57BL/6 mice; HUA induced by potassium oxonate	Hyperuricemia model; insulin signalling assays in cardiomyocytes	HUA ↑ ROS, inhibited IRS‐1/PI3K/Akt signalling, ↓ GLUT4 translocation; NAC reversed effects	[33]
KK‐A^y/Ta diabetic mice vs. C57BL/6J controls	Observational comparison of metabolic parameters	Hyperuricemia correlated with fasting glucose, insulin, HOMA‐IR, triglycerides	[34]
Cultured cardiomyocytes and mouse tissue	HUA exposure ± AMPK activators (metformin, AICAR)	HUA impaired IRS/Akt; AMPK activation attenuated ROS and improved insulin signalling	[35]
~5000 U.S. non‐diabetic adults	Cross‐sectional survey	Highest SUA quartile had ~2‐fold ↑ odds of IR and hyperinsulinemia vs. lowest quartile	[36]
Obese children and adolescents	Observational mediation analysis	SUA partially mediated BMI–HOMA‐IR relationship	[37]
Mendelian randomization (European cohorts)	Genetic instruments for SUA	No causal effect of SUA on fasting insulin; suggests reverse causality possible	[38]
73 asymptomatic hyperuricemic adults	3‐month allopurinol therapy	↓ SUA, ↓ fasting insulin, ↓ HOMA‐IR, ↓ hs‐CRP	[20]
Hyperuricemic patients with renal stones (*n* = 15)	6‐month allopurinol 300 mg/day	↓ fasting insulin and C‐peptide; nonsignificant ↓ in fasting glucose	[39]
Meta‐analysis of 7 RCTs (~503 participants)	Urate‐lowering therapy (allopurinol, febuxostat)	Mean ↓ in fasting insulin (~−1.4 μIU/mL) and HOMA‐IR (~−0.65); no significant effect on fasting glucose	[40]

*Note:* Key experimental, observational, and interventional evidence linking SUA to IR are summarised in the table.

Abbreviations: AMPK, AMP‐activated protein kinase; GLUT4, glucose transporter type 4; HOMA‐IR, homeostasis model assessment of insulin resistance; hs‐CRP, high‐sensitivity C‐reactive protein; HUA, hyperuricemia; IRS‐1, insulin receptor substrate‐1; PI3K, phosphoinositide 3‐kinase; RCT, randomised controlled trial; ROS, reactive oxygen species.

Mechanistic investigations further demonstrate that elevated SUA induces oxidative stress, activates the NLRP3 inflammasome, and decreases NO bioavailability, which in turn impairs insulin‐mediated glucose uptake in muscle and adipose tissues [12]. Genetic models provide additional support for a causal relationship: mice with reduced uricase activity—more closely resembling human UA metabolism—exhibit higher SUA levels and greater susceptibility to diet‐induced insulin resistance [11].

### 5.2 Clinical and Epidemiological Studies

Human studies largely support these experimental observations. Several epidemiological studies demonstrate a positive association between elevated SUA and the risk of IR, T2D, and metabolic syndrome. Research involving prospective cohorts from both the U.S. and Asia indicates that baseline SUA levels can predict the future development of IR and new cases of diabetes, regardless of conventional risk factors like obesity or hypertension [41, 42]. Cross‐sectional studies similarly report that higher SUA levels are associated with various indicators of IR, such as the homeostasis model assessment of IR (HOMA‐IR) [43]. Hyperuricemia often occurs prior to the manifestation of components of metabolic syndrome, implying a contributing role rather than merely a coincidental association [44, 45].

Interventional studies, though more limited, further support the possible causal relationship. Small‐scale randomised and open‐label studies indicate that urate‐lowering medications can enhance insulin sensitivity and endothelial function. Treatment with allopurinol among patients experiencing hyperuricemia and metabolic syndrome led to improvements in HOMA‐IR and increased availability of endothelial NO when compared to placebo [20]. Febuxostat has exhibited similar impacts on insulin sensitivity and inflammatory markers in those suffering from gout or asymptomatic hyperuricemia [7]. In adolescents with recent onset of hypertension and hyperuricemia, allopurinol resulted in reduced SUA and blood pressure, as well as improved insulin sensitivity [46].

Together, these experimental and clinical studies suggest that elevated SUA is not simply a marker of metabolic dysfunction but an active contributor in the development of IR.

Despite this supportive evidence, some counter‐findings introduce uncertainty. Some Mendelian randomization analyses and recent reviews report little or no causal effect of genetically elevated SUA on T2D or related metabolic traits, arguing against a straightforward causal interpretation [6, 47]. These discrepancies may come from residual confounding in population studies such as body weight, diet, alcohol use, kidney function, and socioeconomic status. They may also arise from reverse causation, where insulin resistance raises SUA levels, as well as sex‐specific effects and differences between lifelong genetic elevation and short‐term biochemical changes. Measurement differences, like single SUA measures and varying insulin resistance indices, along with limited power or pleiotropy in genetic tools, also limit the conclusions from Mendelian randomization [48, 49].

Overall, while biologically plausible and supported by several lines of evidence, the causal relationship between SUA and IR in humans remains incompletely resolved. Future studies should prioritise well‐designed randomised trials of urate‐lowering therapies, as well as development of stronger genetic tools to better clarify the direction and causality of this relationship.

## 6 Therapeutic and Translational Implications

Growing evidence suggests that high SUA levels play a significant role in IR and related metabolic disturbances. This has promoted interest in treatment methods that address UA metabolism in conjunction with conventional lifestyle and pharmacologic treatments for metabolic disorders.

### 6.1 Lifestyle Interventions

Adjusting dietary habits remains essential for managing hyperuricemia and IR. Observational and interventional studies consistently show that limiting fructose intake, especially from sugar‐sweetened beverages, and reducing purine‐rich foods, such as organ meats and some seafood, lowers SUA and improves HOMA‐IR, often independent of weight loss [50]. Adopting plant‐based or dietary approaches to stop hypertension (DASH)‐style diets, which are rich in fruits, vegetables, and low‐fat dairy, is associated with lower SUA levels and better metabolic profiles [51]. These dietary changes are connected to reduced production of urate in the liver, decreased de novo lipogenesis, and mitigation of oxidative stress.

Regular physical activity improves insulin‐mediated glucose uptake in skeletal muscle and stimulates renal urate excretion. Observational studies show a negative relationship between exercise levels and both SUA and HOMA‐IR, highlighting two metabolic benefits [42, 52]. Similarly, weight reduction through cutting calories or bariatric surgery lowers SUA and improves IR, indicating the shared mechanisms involved in fat accumulation, urate management, and insulin signalling [19, 53].

### 6.2 Pharmacologic Interventions

Pharmacologic approaches differ in mechanism, approval status, and clinical evidence. Xanthine oxidase (XO) inhibitors, including allopurinol and febuxostat, decrease SUA by blocking UA production. Limited randomised trials and meta‐analyses have shown these medications lower HOMA‐IR and fasting insulin levels in those who are hyperuricemic or at higher risk; however, the results vary and long‐term impacts are still being studied [40, 54].

Sodium‐glucose co‐transporter 2 (SGLT2) inhibitors, used to treat T2D, provide a dual advantage: they lower SUA by facilitating the urinary excretion of UA while also improving insulin sensitivity and glycemic control [55, 56]. Studies also indicate that SGLT2 inhibitors reduce the incidence of gout and may indirectly reduce complications associated with IR [57]. Other potential strategies, like recombinant uricase formulations and dual‐action metabolic drugs, are still being studied and need more clinical validation.

### 6.3 Biomarkers and Risk Stratification

SUA itself serves as a readily available biomarker for identifying individuals who are at risk of developing IR and cardiometabolic diseases. Incorporating SUA measurements into risk‐prediction models alongside fasting insulin, HOMA‐IR, and criteria for metabolic syndrome enhances the early identification of high‐risk individuals [58]. Additional markers, including fractional excretion of UA, UA‐to‐creatinine ratio, and XO activity, could improve risk stratification and inform personalised treatments [7]. From a translational perspective, combining SUA measurements with additional biomarkers such as adipokines, inflammatory cytokines, and oxidative stress indicators may help patients who are likely to benefit from targeted interventions, whether lifestyle‐based or pharmacologic.

Overall, lifestyle and pharmacologic interventions affect IR and metabolic health through pathways mentioned earlier. These include liver glucose production, lipogenesis, oxidative stress, and adipose inflammation. Understanding the strength, type, and basis of evidence helps tailor personalised treatment plans and points out areas that need more research, especially concerning XO inhibitors and emerging urate‐targeted therapies.

## 7 Knowledge Gaps and Future Directions

While there is increasing evidence that SUA plays a role in IR, important mechanistic and translational issues still need to be addressed. Addressing these gaps is vital for improving our understanding of the relationship between UA and insulin signalling and for developing effective clinical interventions.

Unresolved Mechanistic Questions: Despite robust experimental evidence linking SUA to oxidative stress, endothelial dysfunction, inflammation, and mitochondrial impairment, the relative contributions and importance of these pathways are still not fully understood. For instance, it is unclear whether ROS generated by XO are the primary factors driving IR or if the activation of the NLRP3 inflammasome plays an equally important role [17]. The relationship between SUA and adipokines, such as adiponectin, leptin, and resistin, needs clarification as most existing studies show a correlation but do not prove a cause‐and‐effect relationship [19]. Intracellular and extracellular urate might trigger different signalling pathways. However, the ways they are transported and the effects on specific tissues are not well understood [7]. Genetic variations in urate transporters, like SLC2A9 and ABCG2, may modulate IR susceptibility. However, their functional relevance in humans is still unclear. Resolving these mechanisms is crucial for designing precise therapeutic strategies targeting urate‐related metabolic dysfunction.

Need for longitudinal and interventional human studies: much of the epidemiological data linking SUA to IR has been derived from cross‐sectional or short‐term prospective studies. Although meta‐analyses indicate that elevated SUA is a predictor of incident T2D [43], confounding factors like adiposity, dietary habits, and renal function complicate causal conclusions. Mendelian randomization studies have produced varied outcomes, suggesting that genetic factors influencing urate may not consistently translate into metabolic risk [59]. Well‐designed, longitudinal studies with repeated assessments of SUA, insulin sensitivity, and renal function are needed for elucidating temporal associations. Randomised controlled trials assessing urate‐lowering therapies—particularly XO inhibitors and SGLT2 inhibitors—have yielded variable effects on HOMA‐IR and glycemic outcomes [40]. Future trials should include multiethnic populations, standardised metabolic endpoints, and grouping based on baseline SUA, kidney function, and urate transporter genotypes to identify individuals who are most likely to benefit.

Emerging therapeutic targets: Beyond traditional urate‐lowering approaches, pathways involved in inflammasome activation and mitochondrial biogenesis present promising targets for reducing SUA‐induced IR [17]. SGLT2 inhibitors exemplify dual‐action therapies that both enhance urate excretion and improve insulin sensitivity, illustrating the potential for integrated metabolic interventions [57]. Concurrently, systems biology and multi‐omics approaches—integrating metabolomics, proteomics, and genomics—may uncover yet undiscovered networks and biomarkers influenced by UA, facilitating early risk prediction and personalised treatment strategies.

## 8 Conclusions

Accumulating evidence indicates that SUA is not merely a metabolic by‐product but an active modulator of insulin resistance through interconnected mechanisms involving oxidative stress, endothelial dysfunction, inflammation, mitochondrial impairment, and dysregulated adipokine signalling. Experimental and clinical studies consistently demonstrate that high SUA can impair insulin signalling in the liver, skeletal muscle, and adipose tissue, while metabolic disturbances such as obesity and hyperinsulinemia further exacerbate hyperuricemia, creating a reinforcing pathological cycle.

Despite strong biological plausibility, the causal role of SUA in human insulin resistance is still not fully understood. There are important gaps in knowledge, such as how much individual pathways contribute, including the roles of XO‐derived ROS versus NLRP3 inflammasome activation. The effects of urate inside cells compared to outside cells also need more clarity, as does the impact of genetic differences in urate transporters. Furthermore, inconsistencies among observational studies, Mendelian randomization analyses, and intervention trials highlight the need for more rigorous study designs.

Future research should focus on well‐designed longitudinal studies and randomised controlled trials that consider baseline SUA, kidney function, and genetic background. Integrating multi‐omics approaches with mechanistic and clinical studies will be crucial for understanding causality and identifying individuals who are most likely to benefit from urate‐targeted metabolic interventions.

## Author Contributions

**Nurshad Ali:** conceptualization, writing – original draft, writing – review and editing.

## Conflicts of Interest

The author declares no conflicts of interest.

## Data Availability

Data sharing not applicable to this article as no datasets were generated or analysed during the current study.
